# The Manx shearwater (*Puffinus puffinus*) as a candidate sentinel of Atlantic Ocean health

**DOI:** 10.1186/2046-9063-10-6

**Published:** 2014-09-01

**Authors:** Maíra Duarte Cardoso, Jailson Fulgencio de Moura, Davi C Tavares, Rodrigo A Gonçalves, Fernanda I Colabuono, Emily M Roges, Roberta Laine de Souza, Dalia Dos Prazeres Rodrigues, Rosalinda C Montone, Salvatore Siciliano

**Affiliations:** 1 Programa de Pós-Graduação em Saúde Pública e Meio Ambiente, ENSP/Fiocruz, Rua Leopoldo Bulhões, 1480, Manguinhos, Rio de Janeiro 21041-210, RJ, Brasil; 2 Systems Ecology, Leibniz Center for Tropical Marine Ecology (ZMT), Fahrenheitstrasse 6, 28359 Bremen, Germany; 3 Departamento de Endemias Samuel Pessoa – DENSP & Grupo de Estudos de Mamíferos Marinhos da Região dos Lagos – GEMM-Lagos, Escola Nacional de Saúde Pública /FICORUZ, Rua Leopoldo Bulhões, 1.480, 6° andar, Sala 611, Manguinhos, Rio de Janeiro 21041-210, RJ, Brasil; 4 Departamento de Química, Pontifícia Universidade Católica do Rio de Janeiro, Rua Marquês de São Vicente, 225, Gávea, Rio de Janeiro 22453-900, RJ, Brasil; 5 Universidade de São Paulo, Instituto Oceanográfico, Praça do Oceanográfico 191, Cidade Universitária, São Paulo 05508-120, SP, Brasil; 6 Instituto Oswaldo Cruz/FIOCRUZ, Laboratório de Referência Nacional de Enteroinfecções Bacterianas, Av. Brasil, 4365, Manguinhos, Rio de Janeiro 21040-360, RJ, Brasil

**Keywords:** *Puffinus puffinus*, Brazil, Sentinel, Metal, Organochlorines, *Vibrio*, *Aeromonas*

## Abstract

**Introduction:**

Seabirds have been historically used to monitor environmental contamination. The aim of the present study was to test the suitability of a species belonging to the Procellariiformes group, the Manx shearwater, *Puffinus puffinus*, as a sentinel of environmental health, by determining contaminant levels (trace metals and organochlorine compounds) from carcass tissues and by isolating *Vibrio* spp. and *Aeromonas* spp. from live specimens. To this end, 35 *Puffinus puffinus* carcasses wrecked on the north-central coast of the state of Rio de Janeiro, Brazil, and two carcasses recovered in Aracruz, on the coast of the state of Espírito Santo, Brazil, were sampled, and fragments of muscle and hepatic tissues were collected for contaminant analyses. Swabs from eleven birds found alive at the north-central coast of Rio de Janeiro were collected for isolation of the aforementioned bacteria.

**Results:**

The average concentration in dry weight (dw) of the trace metals were: mercury 7.19 mg kg^-1^(liver) and 1.23 mg kg^-1^ (muscle); selenium 34.66 mg kg^-1^ (liver) and 7.98 mg kg^-1^ (muscle); cadmium 22.33 mg kg^-1^ (liver) and 1.11 mg kg^-1^ (muscle); and lead, 0.1 mg kg^--1^ (liver) and 0.16 mg kg^-1^ (muscle). Organochlorine compounds were detected in all specimens, and hexachlorbiphenyls, heptachlorbiphenyls and DDTs presented the highest levels. Regarding microbiological contamination, bacteria from the *Vibrio* genus were isolated from 91% of the analyzed specimens. *Vibrio harveyi* was the predominant species. Bacteria from the *Aeromonas* genus were isolated from 18% of the specimens. *Aeromonas sobria* was the only identified species.

**Conclusions:**

The results indicate that *Puffinus puffinus* seems to be a competent ocean health sentinel. Therefore, the monitoring of contaminant levels and the isolation of public health interest bacteria should proceed in order to consolidate this species importance as a sentinel.

## Introduction

Oceans cover approximately 70% of the earth surface [1] and about 60% of the human population lives in coastal areas. Many of these populations depend on the ocean for their subsistence [2].

As environmental degradation accelerates, science has increasingly focused on the influence of the environment on human health. Environmental degradation has direct impacts on life quality and health conditions of the human population [3]. Consequently, ocean processes, which are influenced by human activity, have important public health implications [4,5].

Some substances, such as persistent organic pollutants (POPs), polycyclic aromatic hydrocarbon (PAHs) and toxic metals show negative impacts on the health of humans and other animals and also on the oceans themselves [5-8]. The same is true with regard to pathogenic microorganisms, especially those autochthonous of marine environments, like bacteria from the *Vibrio* and *Aeromonas* genera [5,7].

As these substances reach the marine environment, they impact the biota in a negative manner [9]. Metals are naturally present in marine environments, and they only become toxic when their concentration levels are increased beyond a certain point [10]. Many organochlorine compounds are synthetic and reach the environment mainly by anthropic action [9]. These two classes of pollutants are persistent in the marine environment and are capable of bioaccumulation and biomagnification in the marine food web [10].

There is a need for coastal countries to develop ocean monitoring strategies [11] and one way of doing this is through the use of sentinel species [12]. These species are capable of accumulating pollutants in their organisms without significant adverse effects and are used to measure the amount of bioavailable pollutants [13]. Seabirds have been historically used as sentinels because they are well-known, conspicuous, ubiquitous, abundant, large, long-living, well-liked by people, and, most importantly, they are top predators in the food chain. This is important, since the determination of pollutants that are capable of bioaccumulation and biomagnification is most adequate in higher level organisms [14-16].

In this context, the present study suggests the use of the Manx shearwater (*Puffinus puffinus*) as a sentinel of Atlantic Ocean health, as this species fulfills the requisites of a good sentinel species [17]. The oldest specimen ever recorded was over fifty years old [18] and a 31-year-old specimen was recovered from the Brazilian coast in 2009 (GEMM-Lagos, unpublished data). Manx shearwater colonies are located in the North Atlantic Ocean, mostly in the United Kingdom [17]. During the northern winter, the Manx shearwater migrates to the South Atlantic Ocean, with the Brazilian coast as its main destination [17,19]. The objection that a migratory species should not be used for this purpose, as they are not specific to that particular environment, does not stand when the goal is to gather data at a large scale [14].

Thus, the aim of the present study was to test the usefulness of *P. puffinus* as a sentinel of environmental health, by determining contaminant levels (trace metals and organochlorine compounds) from carcass tissues and by isolating *Vibrio* spp. and *Aeromonas* spp. from live specimens.

## Results and discussion

### Biometric data

The length of the carcasses collected in this study was of 32.88 ± 0.49 (mean ± standard deviation), ranging from 30 to 36 cm. The wingspan was of 72.24 ± 0.52 (mean ± standard deviation), ranging from 69 to 76 cm.

### Metal analyses

Element concentrations are presented in Table 1, as mean ± standard deviation (SD) and range (min-max) on a dry weight basis. Tables 2 and 3 show the values obtained and the recovery levels of the certified reference material analyses. The method limits of quantification for muscle tissue were 0.02 mg.kg^-1^ for Hg; 0.072 mg.kg^-1^ for Se; 0.071 mg.kg^-1^ for Cd; and 0.097 mg.kg^-1^ for Pb. The limits for hepatic tissue were 0.02 mg.kg^-1^ for Hg; 0.59 mg.kg^-1^ for Se; 0.28 mg.kg^-1^ for Cd; and 0.009 mg.kg^-1^ for Pb.

**Table 1:** **Metal concentrations tissues of in *Puffinus puffinus *** **wrecked at study area**

**Tissue**	**[Hg] (dw) (mg.kg**^**-1**^**)**	**[Se] (dw) (mg.kg**^**-1**^**)**	**[Cd] (dw) (mg.kg**^**-1**^**)**	**[Pb] (dw) (mg.kg**^**-1**^**)**
**Hepatic (n = 20)**	7.19 ± 3.37 (1.16 – 14.22)	34.66 ± 20.14 (10.56 – 75.20)	22.33 ± 25.46 (2.31 – 113.01)	0.1 ± 0.06 (0.036 – 0.28)
**Muscle (n = 37)**	1.23 ± 0.53 (0.47 – 2.31)	7.98 ± 3.68 (3.17 – 19.01)	1.11 ± 1.72 (<LQ^*^ – 8.94)	0.16 ± 0.09 (<LQ^*^ – 0.43)

*< LQ = below the limits of quantification.

Detailed legend: Metal concentrations tissues of in *Puffinus puffinus* wrecked at study area, from 2005 to 2011.

**Table 2:** Analyses and recovery of the certified reference materials (DORM-2)

**Metal**	**Reference value DORM-2 (mg.kg**^**-1**^**)**	**Mean of obtained values (mg.kg**^**-1**^**)**	**Recovery (%)**	**n**
**Cd**	0.043 ± 0.008	0.04	97%	3
**Hg**	4.64 ± 0.26	4.83	104%	3
**Pb**	0.065 ± 0.007	0.07	104%	3
**Se**	1.4 ± 0.09	1.39	99%	3

**Table 3:** Analyses and recovery of the certified reference materials (DOLT-3)

**Metal**	**Reference value DORM-2 (mg.kg-1)**	**Mean of obtained values (mg.kg-1)**	**Recovery (%)**	**n**
**Cd**	19.4 ± 0.6	17.45	90%	3
**Hg**	3.37 ± 0.14	3.17	94%	3
**Pb**	0.319 ± 0.045	0.29	92%	3
**Se**	7.06 ± 0.48	6.98	99%	3

The results of this study are in accordance with worldwide literature. Mean Hg concentrations in *P. puffinus* hepatic tissue were slightly higher than those reported by Dale *et al.*[20] but somewhat lower than those reported by Osborn *et al.*[21]. The means in muscle tissue were somewhat higher than the results by Osborn *et al.*[21]. These comparisons suggest that mercury concentrations in this species have not varied much from the 1970s to the present day.

Mean hepatic Cd concentrations in this study are higher than those described by Osborn *et al.*[21] and Garcia [22], but this is mainly due to outliers. If the median (16.25 mg.kg^-1^) had been used instead of the means, the results would have been very close to those reported by the aforementioned studies. In muscle tissue, Cd concentrations were lower than those described by Osborn *et al.*[21] and Garcia [22].

Comparing the results of this study with others involving different *Puffinus* genus species with similar habits, lower metal concentrations are usually reported in species that breed in the southern hemisphere (*P. gravis*, *P. assimilis* and *P. griseus*) [23-26]. It is possible that the North Atlantic Ocean, where *P. puffinus* colonies are located, shows higher metal contamination rates, due to earlier and more intense industrialization, but the differences observed could also be due to different diets within the species [23].

On the other hand, Cd concentrations reported by Muirhead & Furness [23] in *P. gravis* and *P. assimilis* are almost twice those observed in the present study. Garcia [22] also reported higher concentrations in *P. gravis* tissues. The concentration of this metal tends to be higher in species whose diet consists of a high amount of cephalopods [23], since these animals are important transfer vectors of cadmium in the food chain [27,28]. Cephalopods are important prey in *P. assimilis*, *P. gravis* and *P. puffinus* diets [17,23,29], so it is possible that the species studied by Muirhead & Furness [23] and Garcia [22] fed on cephalopods that accumulate higher cadmium concentrations, or simply fed on a higher amount of cephalopods, as compared to the *P. puffinus* specimens analyzed in this study. Differences in the diet of this species can be seen in the study by Petry *et al.*[29]. Another hypothesis is that the specimens of the previously cited studies were older than those used in the present study, since cadmium tends to accumulate over the years [30].

Pb concentrations reported in studies involving *P. gravis* and *P. griseus* were very low, in accordance to the present study [25,26]. This is due to the biodilution that the element seems to undergo in the marine food chain [31,32].

It is important to highlight that most cephalopod species and all the fish species cited as Manx shearwater food sources in several studies, such as *Ammodytes tobianus*, *Mallotus villosus*, *Paralonchurus brasiliensis*, *Clupea* genus juveniles and juvenile squid from the Ommastrephidae family [17,29,33], are also consumed by humans, indicating that humans can be exposed to these contaminants through their diet.

#### Concentrations of the same metal in hepatic and muscle tissues

Spearman’s rank correlation coefficient was used in order to verify the strength of the association between the concentration of an element in the liver and the concentration of the same element in the muscle. The classification of the strength of the association followed the scale described by Bryman & Cramer [34].

**Figure 1:**
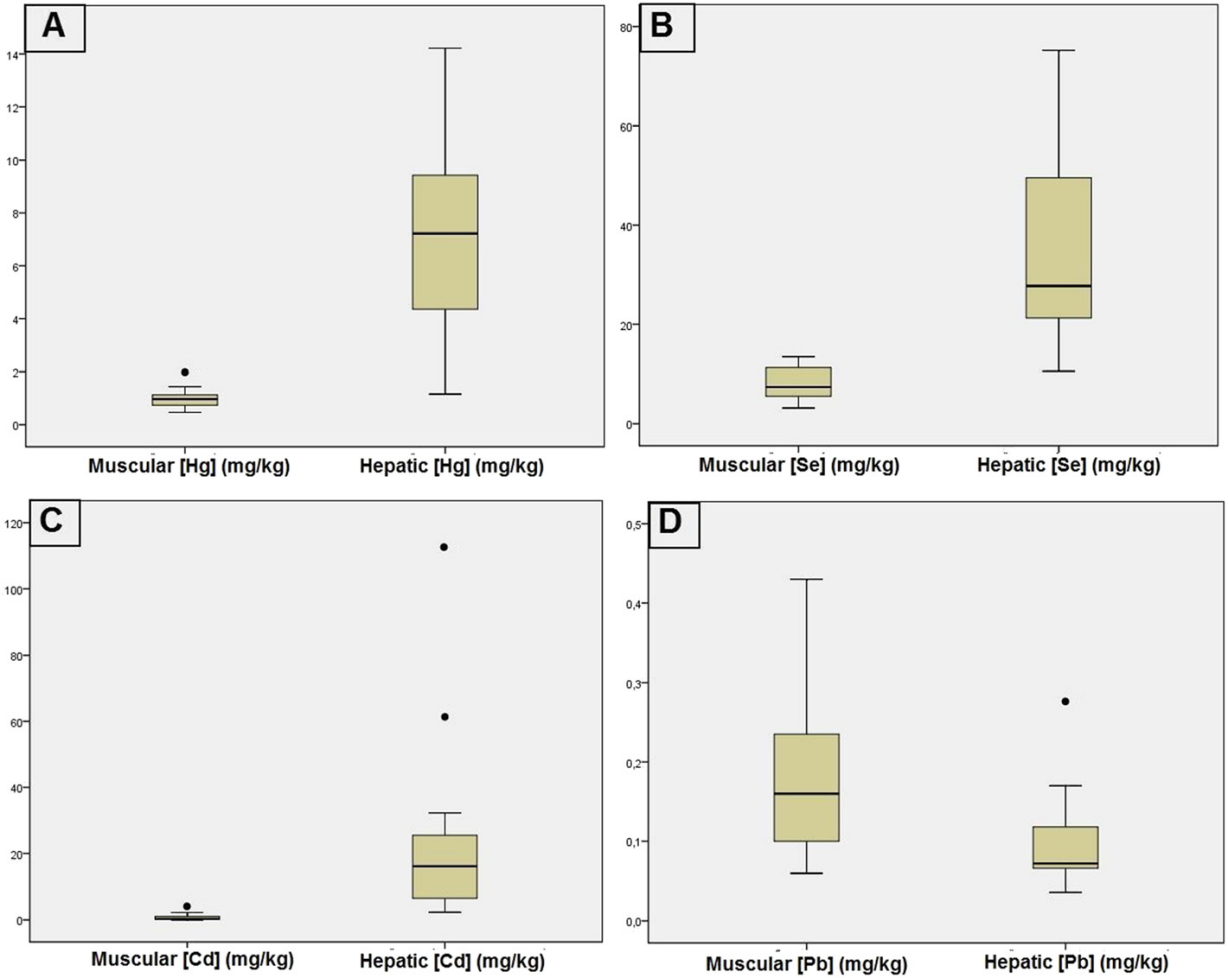
**Differences between muscle and hepatic metal concentrations in *Puffinus puffinus *tissues.** Detailed legend: Differences between muscle (n = 37) and hepatic (n = 20) metal concentrations in *Puffinus puffinus* wrecked at the study area, from 2005 to 2011. **A** = [Hg]; **B** = [Se]; **C** = [Cd]; **D** = [Pb].

Moderate positive correlations were found between hepatic and muscle Hg (ρ = 0.50) and between hepatic and muscle Se (ρ = 0.48). A strong positive correlation was found between hepatic and muscle Cd (ρ = 0.81), and a weak positive correlation, with no statistical significance, was found between hepatic and muscle Pb (ρ = 0.29).The differences in element concentrations in both tissues can be seen in Figure 1, where all elements, except for Pb, show the same pattern, with higher concentrations in hepatic tissue when compared to muscle.

In birds, 90% of the existing cadmium is accumulated in the liver and kidneys, the latter being the main site of cadmium toxicity, since kidneys do not resist the toxic effects of cadmium as well as the liver. For this reason, hepatic tissue is considered the best locus to monitor cadmium exposition. It is also the best choice for monitoring mercury and selenium exposure [35]. This is possibly the reason why, in the present study, Cd levels in the liver were always higher than in muscle.

In this study, Pb behaved differently from the other analyzed metals, but it is important to note that Pb levels were very low and the differences between muscle and liver concentrations were very small. In birds, this element accumulates primarily in the bones and among the soft tissues, with the primary accumulation site being the kidneys [35,36]. It is known that lead can occupy the binding sites of calcium [37], so this is possibly the reason why, in the present study, the element was found at its highest concentration in muscle tissues.

#### Interelemental relationships

The correlations between the elements were tested by the Spearman’s rank correlation coefficient. Very weak positive correlations, with no statistical significance, were found between Hg and Se (ρ = 0.05) and Se and Cd (ρ = 0.04), both in muscle tissue; and also between Cd and Pb (ρ = 0.16) and Hg and Pb (ρ = 0.10), both in hepatic tissue. Very weak negative correlations, with no statistical significance, were found between Hg and Cd (ρ = -0.04) and Hg and Pb (ρ = -0.06), both in muscle tissue. Weak positive correlations, with no statistical significance, were found between Se and Pb (ρ = 0.20) and Cd and Pb (ρ = 0.20), both in muscle tissue; and the same was observed between Se and Pb in hepatic tissue (ρ = 0.20).

However, some statistically significant correlations were found, such as the moderate positive correlations found between Hg and Se (ρ = 0.46), Hg and Cd (ρ = 0.52) and Se and Cd (ρ = 0.50), all in hepatic tissue.

Selenium and mercury tend to co-accumulate in bird livers, where selenium acts in reducing mercury toxicity [38-40], although this detoxifying process is not well elucidated. Some possible mechanisms involved in this are the redistribution of mercury throughout the organism in the presence of selenium; competition between the two elements for binding sites; formation of complexes between these elements; conversion of toxic forms of mercury into less toxic forms, such as the demethylation of methyl mercury by selenium; and prevention of oxidative stress caused by mercury [38,41,42].

In marine mammals, a molar ratio of 1:1 between Hg and Se is found when Se is being used to detoxify Hg and vice-versa [43]. In birds, this ratio is almost never found [38,43,44], as in the present study, where a 1:5 ratio was found. This is possibly the reason why the correlation between Hg and Se found here is moderate, and not strong or very strong, as has been reported in several studies involving marine mammals [45-47], since this protection mechanism seems to be inherent to marine mammals [43]. Other studies involving aquatic birds have found a larger quantity of selenium relative to mercury, precluding the 1:1 molar ratio, as in the present study [42,44,48].

Current literature has also suggested a correlation between Se and Cd in marine birds [30]. Besides detoxifying mercury, selenium can also detoxify cadmium in these organisms, probably by altering the availability of this metal [30].

Selenium seems to be highly effective against damage caused by cadmium and also against methyl mercury and inorganic mercury toxicity, and seems to have very little effect against lead toxicity [41]. This is possibly the reason why, in the present study, Se, Cd and Hg showed correlations, while Pb did not.

### Organochlorine compounds analyses

Organochlorine compound concentrations are presented in Table 4, as a sum of each group and range (min-max) in ng.g^-1^, on a dry weight basis.

**Table 4:** **Concentrations of organochlorine compounds in tissues of *Puffinus puffinus *** **wrecked at study area**

**Compounds**	**Results (dw) (ng.g**^**-1**^**) (n = 13)**
**ΣHCHs**^**(a)**^	<LQ^*^
**HCB**	16.7 (5.97 – 34.3)
**ΣChlordanes**^**(b)**^	9.59 (<LQ^*^ – 20.8)
**ΣDrins**^**(c)**^	31.39 (<LQ^*^ – 65.2)
**ΣDDTs**^**(d)**^	193.42 (<LQ^*^ – 626.0)
**Endosulfan II**	<LQ^*^
**Metoxychlor**	<LQ^*^
**Mirex**	8.94 (<LQ^*^ – 36.9)
**ΣPCBs**^**(e)**^	729.16 (<LQ^*^ – 632.0)

^(a)^ = sum of means of α-, β-, γ- and δ-isomer; ^(b)^ = sum of means of γ-, α –chlordane, oxychlordane, heptachlor and heptachlor epoxide A and B; ^(c)^ = sum of means of aldrin, endrin, isodrin and dieldrin; ^(d)^ = sum of means of pp’ DDT, op’ DDT, pp’ DDE, op’ DDE, pp’ DDD and op’ DDD; ^(e)^ = sum of means of 51 congeners; ^*^< LQ = below limits of quantification.

Detailed legend: Concentrations of organochlorine compounds in tissues of *Puffinus puffinus* wrecked at study area, from 2005 to 2011.

In this study, organochlorines were found in all specimens. Among the OCPs, the DDT group predominated, especially pp’ DDE. Among the PCBs, there was a predominance of hexachlorbiphenyls and heptachlorbiphenyls.

The standard deviation for each compound was extremely high, which indicates great individual variation in the contaminant burden. This variation is expected in long-living birds with delayed sexual maturity, even among specimens at the same maturity stage. This can be related to distribution, migration, diet or age of the individuals [49]. The fact that *P. puffinus* is a migratory species must also be taken into account, which makes it likely that different individuals have different diets depending on the places visited throughout the migration period, reflecting the contamination of breeding and migration sites [50].

In the DDT group, pp’ DDE was predominant and found in greater quantities among all the OCPs, in accordance with other studies [49,51]. This compound is usually found in the tissues of top predators due to its stability, bioconcentration and bioaccumulation [52]. In birds, DDT is metabolized to DDD and DDE, and DDD to DDE and DDMU, therefore it is normal that DDT and DDD concentrations decrease while DDE increases [53].

HCHs were not detected in any sample, probably due to the rapid metabolization and elimination that these compounds undergo in birds [54].

Oxychlordane is a metabolite belonging to the chlordanes group. It is persistent and presents difficult biotransformation in seabirds. It is therefore accumulated in greater amount in these organisms than other compounds of the group [49,55]. However, in the present study, this compound was only detected in five specimens and in very low concentrations.

From the drins group, only dieldrin was detected. This can be due to the rapid metabolization of aldrin into dieldrin and to the storage of this form in animals [56]. Isodrin is an aldrin isomer and endrin may not have been detected due to its rapid metabolization and excretion [57].

Endosulfan and methoxychlor are also metabolized and excreted fairly quickly [58,59], which may explain their absence in the analyzed tissues. In contrast, HCB and mirex, compounds of great persistance in the environment and biota [60,61], were present in the specimens analyzed in this study.

**Figure 2:**
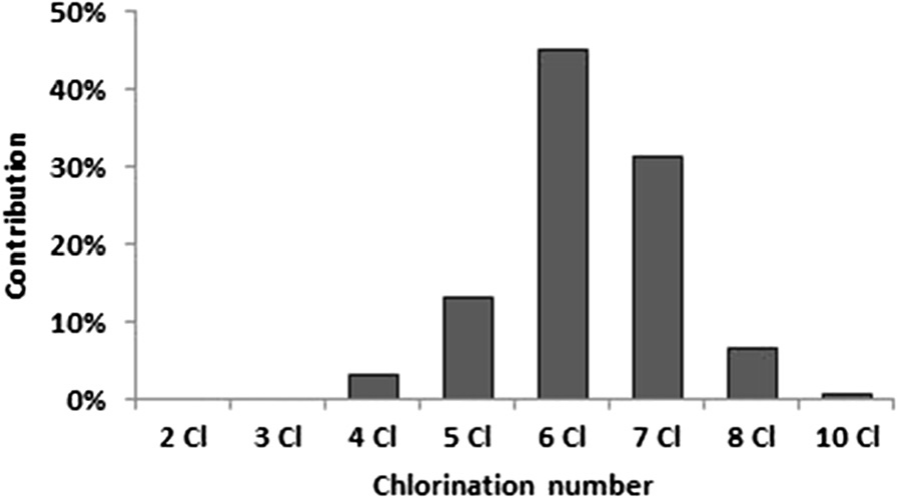
**Mean relative contribution of PCB homologs in *Puffinus puffinus *muscle.** Detailed legend: Mean relative contribution of PCB homologs from two to ten chlorine atoms found in *Puffinus puffinus* muscle.

With regard to PCBs, there was a predominance of hexachlorbiphenyls and heptachlorbiphenyls (Figure 2). This is due to the fact that birds tend to metabolize and excrete low molecular weight PCBs congeners and accumulate high weight congeners that present a higher degree of halogenation [62].

In another study involving *P. puffinus*, in addition to other Procellariiformes, conducted at Rio Grande do Sul, Brazil, by Colabuono *et al.*[49], a similar pattern of contamination was found in tissues: a predominance of pentachlorobiphenyls, hexachlorobiphenyls and heptachlorobiphenyls among PCBs and a predominance of DDTs, especially DDE, among the OCPs. A predominance of DDE has also been reported by Bourne & Bogan [51], in a study involving seabirds from the North Atlantic, including the Manx shearwater. However, in contrast to the present study and to Colabuono *et al.*[49], uniformity in organochlorine levels was detected in the analyzed bird tissues.

In another study involving this species, higher levels of pp’ DDE and lower levels of PCBs were reported in tissues of specimens from the Mediterranean and from the Black Sea [63]. Another study conducted in Brazil detected all of the analyzed PCBs congeners and, in contrast to the present study, pentachlorobiphenyls were predominant and levels of hexa- and heptacholobiphenyls were low [64].

### Microbiological analyses

Bacteria from the *Vibrio* genus were detected in 90% of the analyzed specimens (n = 11), with *V. harveyi* being the most frequent species, followed by *Vibrio mediterranei* and *V. parahaemolyticus*, each one present in 19% of the cases. Other species were also isolated: *V. fluvialis* (7%), *V. fisheri* (7%), *V. cincinnatiensis* (7%), *V. orientalis* (4%), *V. cholerae* non-01/non-0139 (4%), *V. gazogenes* (4%) and *V. alginolyticus* (4%). In 3% of the cases, classification by species was not possible.

Bacteria from the *Aeromonas* genus were isolated in only 18% of the specimens (n = 11) and in most cases (67%), classification by species was not possible. In the remaining cases (33%), only one species was isolated: *Aeromonas sobria*.

*Vibrio anguillarum* and *V. tapetis* are the bacteria most frequently related to diseases in aquatic animals [65]. None of these was isolated in the present study. Some species are known to cause diseases in both animals and humans, being potential zoonoses agents, such as *V. alginolyticus*, *V. harveyi*, *V. cholerae*, *V. fluvialis*, *V. furnissii*, V. mimicus, *V. metschnikovii*, *V. parahaemolyticus* and *V. vulnificus*[65]. Attention should be paid to the fact that five of these species were isolated in the present study, considering that an important infection route for animals and humans is through contaminated seafood [66,67].

Some coincident species were isolated in studies involving aquatic birds in the USA [68,69], Brazil [70], Japan [66] and England [71].

Migratory birds, such as the Manx shearwater, are important dispersers of micro-organisms. Additionally, when migrating, these birds tend to meet at certain locations, which facilitates interindividual and interspecies transmission, even more so since migration stress contributes in decreasing resistance to infection [72]. An important pathogen that can be carried by migratory seabirds is *Vibrio cholerae*[67,72], which can allow for cholera outbreaks in regions distant from endemic areas [72]. In this study, as in the study of Lee *et al.*[71], and as was the case of most birds studied by Ogg *et al.*[68], the *Vibrio cholerae* isolated was not from the O1 or O139 serogroups, which are responsible for cholera disease [73].

In some *Vibrio* species, such as *V. vulnificus*, *V. parahaemolyticus* and *V. cholera*, survival is related to water temperature, with most infections taking place during summer, through the consumption of contaminated seafood or through contact of contaminated water with wounds [65,66,71]. With this in mind, some studies [66,71] were able to isolate these species in aquatic birds even during winter, when the frequency of isolation in water was low, which demonstrates that these bacteria can multiply in birds even when environmental conditions are not favorable and that the survival of this species in the gastrointestinal tract by just a few days is already sufficient to disperse those microorganisms throughout large distances [71].

The study area (north-central coast of Rio de Janeiro) is under influence of the Cabo Frio upwelling, which causes a decrease in water temperatures during spring and summer, due to an outcropping of cold, deep and nutrient-rich waters (South Atlantic Central Water, SACW) [74]. From September to April, the surface water temperature rarely exceeds 18°C, and in deeper waters, it is often below 15°C [74,75]. Coincidentally, this is the period when the Manx shearwater is present in the Brazilian coast. Therefore, the species may be an important *Vibrio* spp. carrier when the water temperature is low.

With regard to *Aeromonas* spp., some species also depend on warmer waters to survive, as most isolations and cases of gastroenteritis caused by this genus also occur during summer [76-78]. This fact suggests that the hypothesis that waterfowl are sponsors for these microorganisms during periods of unfavorable environmental temperature can also be applied here, and the period of the influence of the upwelling on the study area should be taken into account.

In a study regarding the source of diarrhea infections caused by *Aeromonas* spp., Moyer [77] reported that some patients may have been contaminated by the ingestion of bivalves and by fishing and swimming in untreated waters. In addition, one of the patients worked at a fish market. These forms of infection indicate the importance of aquatic animals in the transmission of diseases caused by *Aeromonas* spp., and aquatic birds are among those most related as carrying different species from the genus in their gastrointestinal tract [79].

In two studies conducted in Canada, Lévesque *et al.*[80,81] isolated high concentrations of *Aeromonas* spp. from ring-billed gulls feces. These studies emphasize the importance of monitoring these bacteria, as birds can contaminate recreational waters through feces, which may lead to contamination of humans by means of contact between wounds with water or through the ingestion of contaminated seafood [80,81].

## Conclusions

The present study leads to the conclusion that Manx shearwater seems to be an effective sentinel of Atlantic Ocean health, since different types of chemical contaminants could be detected in their tissues and common species of bacteria from aquatic environments could be isolated from swabs collected from this species.

In the present study, metal levels were in accordance with other studies involving this species. The same occurred concerning the pattern of contamination of organochlorine compounds. The results reflect the environmental contamination in breeding sites and throughout the migration route.

As described, *P.* puffinus feeds on fish species and cephalopods which are also consumed by humans, which highlights the contribution of this study to the public health field. It is worth emphasizing the importance of beach monitoring activities in an effort to prevent contamination.

It is recommended that contaminant levels and the frequency of micro-organisms isolation continue to be monitored in this species and in others, in order to evaluate a possible increase in environmental degradation. It is also important to continue studying the species in order to consolidate the Manx shearwater as a sentinel species.

## Methods

For the purposes of this study, between 2005 and 2011, thirty-five carcasses of wrecked Manx shearwater were collected during beach monitoring at the north-central coast of the state of Rio de Janeiro (from Saquarema – south, 22°55’12”S; 42°30’37”W – to São Francisco do Itabapoana – north, 21°18’07”S; 40°57’4”W) and two carcasses were collected at Aracruz (19°49’13”S; 40°16’24”W), in the state of Espírito Santo, Brazil. The carcasses were measured, necropsied and fragments of hepatic and muscle tissues were collected and stored at -20°C for contaminant analyses. For the metal analyses, 37 muscle samples and 20 liver samples were used. For the organochlorine compounds analyses, 13 muscle samples were used. Most carcasses were found in 2010, a year of severe mortality for this species on the Southeastern coast of Brazil.

Furthermore, from 2009 to 2012, cloaca, oral, ocular and tracheal swabs were collected from eleven specimens found alive on beaches of the north-central coast of Rio de Janeiro (from Saquarema to São Francisco do Itabapoana) for the analysis of bacteria from the *Vibrio* and *Aeromonas* genera.

### Analytical methods for metal analyses

The determination of cadmium (Cd), lead (Pb) and selenium (Se) levels was performed by inductively coupled plasma mass spectrometry (ICP-MS). The determination of mercury (Hg) levels was performed by cold vapor atomic absorption spectrometry (CV-AAS).

Samples were defrosted and homogenized with a food microprocessor (HC31 Black & Decker). For Cd, Pb and Se analyses, approximately 0.5 g of each muscle sample, in triplicate, and approximately 0.1 g of each liver sample in duplicate were digested with 5 mL and 1 mL of nitric acid (HNO_3_) (Vetec), respectively, in a heating block (Quimis) (80°C), until total dissolution. Three procedural blanks were prepared with each sample batch in the heating block. Ultra-pure water (obtained from Master All water purificator, Gehaka) was added until a final volume of 50 mL was reached. Metal concentrations were determined using an ICP-MS 7500 Series (Agilent Technologies).

For the Hg analyses, approximately 0.5 g of each muscle sample and approximately 0.1 g of each liver sample, both in duplicate, were digested with 5 mL of a sulphuric-nitric acid mixture (HNO3/H2SO4/V2O5) (Vetec) in a heating block (80°C), until total dissolution. The procedural blanks were performed in the same manner applied to the previous analyses. The samples and the blanks were then cooled and 5 mL of KMnO_4_ 5% (Vetec) were added. The purpose of this process was to ensure that mercury remained in the sample until reading was performed. At this moment, 1 mL of hydroxylamine (Vetec) was added and then ultra-pure water, until a final volume of 50 mL was reached. Finally, concentrations were determined using a 3300 spectrometer (Perkin Elmer).

The accuracy of the analytical methods was ensured by the use of Merck certified material (Hg, Cd, Pb and Se Titrisol Standard Solutions) and the quality of the methods used was ensured by the use of National Research Council of Canada certified reference materials (DORM-2 - Dogfish Muscle Certified Reference Material for Trace Metals; and DOLT-3 - Dogfish Liver Certified Reference Material for Trace Metals), analyzed in parallel, in triplicate, with average recovery ranging from 90% to 104%.

Samples were analyzed as wet weight and, subsequently, aliquots of all samples were weighed and dried in a 315 SE oven (Fanem) until constant weight. This procedure made it possible to obtain humidity factors, which were applied to the results in order to convert them into dry weight.

The limits of quantification (LQ) were provided by equipment software, using the formula: LQ = [10*(SDbr)]/S, where SDbr is the standard deviation from 10 blank readings, and S is the inclination of the calibration curve. This formula provides the instrumental LQ. In order to calculate the final LQ, this formula was multiplied by the dilution factor.

### Analytical methods for organochlorine compounds analyses

The organochlorine pesticides (OCPs) analyzed in this study were: hexachlorocyclohexanes (HCH) (α-, β- , γ- and δ-isomer), hexachlorobenzene (HCB), heptachlor, heptachlor epoxide A and B, chlordanes (oxychlordane, α- and γ- chlordane), drins (aldrin, isodrin, dieldrin, endrin), dichlorodiphenyltrichloroethane (op’ DDT, pp’ DDT) and its metabolites, dichlorodiphenyldichloroethylene (op’ DDE, pp’ DDE) and dichlorodiphenyldichloroethane (op’ DDD, pp’ DDD), endosulfan II, methoxychlor and mirex. The analyzed polychlorinated biphenyls (PCBs) congeners were the following IUPAC numbers: 8, 28, 31, 33, 44, 49, 52, 56/60, 66, 70, 74, 77, 81, 87, 95, 97, 99, 101, 105, 110, 114, 118, 123, 126, 128, 132, 138, 141, 149, 151, 153, 156, 157, 158, 167, 169, 170, 174, 177, 180, 183, 187, 189, 194, 195, 203, 206 and 209.

The analytical procedure followed the protocol described by MacLeod *et al.* (1986) [82], with minor modifications, as described by Colabuono *et al.* (2012) [49].

At first, 2,2’,4,5’,6-pentachlorobiphenyl (PCB 103) and 2,2’,3,3’,4,5,5’,6-octachlorobiphenyl (PCB 198) were added to all samples, blanks and reference material as surrogates for OCPs and PCBs. Then, approximately 2.5 g of each lyophilized muscle sample were extracted in a Soxhlet apparatus for 8 h using 80 mL of n-hexane and methylene chloride (1:1, v/v). The determination of extractable lipids was made by gravimetric analyses. The extracts were cleaned-up through the use of column chromatography with 8 g of silica and 16 g of alumina, both 5% water deactivated, eluted with 80 mL of n-hexane and methylene chloride (1:1, v/v). The fraction was purified once again, in order to remove lipid excess, now through high-performance liquid chromatography (HPLC), using methylene chloride as eluent with a flow of 5 mL min^-1^. The extract was concentrated to a volume of 0.9 mL in hexane. The internal standard 2,4,5,6-tetrachlorometaxylene (TCMX) was added before the gas chromatographic analysis was undertaken and a procedural blank was included in the set of samples.

Identification and quantification analyses of the organochlorine pesticides were performed with a 6890 N gas chromatograph with an electron capture detector (GC-ECD) (Agilent Technologies), using a 30 m × 0.25 mm i.d. capillary column coated with 5% phenyl-substituted dimethylpolysiloxane phase (0.5 μm film thickness). Automatic splitless injections of 2 μL were applied and the total purge rate was adjusted to 50 mL min^-1^. The carrier gas used was hydrogen (constant pressure of 40 kPa at 100°C), and the makeup gas was nitrogen, at a rate of 60 mL min^-1^. Injector and detector temperatures were 280°C and 320°C, respectively. Oven temperature was programmed as follows: 70°C for 1 min, raised at 40°C.min^-1^ until 170°C, then raised at 1.5°C.min^-1^ until 230°C (held for 1 min), and at 20°C.min^-1^ until 300°C with a final hold of 5 min.

The quantitative PCBs analyses were performed by a 5973 N gas chromatograph coupled to a mass spectrometer (GC–MS) (Agilent Technologies), in a selected ion mode (SIM 70 eV), using a 30 m × 0.25 mm i.d. capillary column coated with 5% phenyl-substituted dimethylpolysiloxane phase (0.25 μm film thickness). Injections were made with 1 μL in automatic splitless mode. The carrier gas was helium (constant flow of 1.1 mL min^-1^). The interface, source and quadrupole temperatures were 280°C, 300°C and 200°C, respectively. Oven temperature was programmed as follows: 75°C for 3 min, raised at 15°C.min^-1^ until 150°C, then raised at 2.0°C.min^-1^, until 260°C and at 20°C.min^-1^, until 300°C with a final hold of 10 min.

The analytical methodology was validated through the use of a standard reference (SRM 1945 – organics in whale blubber), from the National Institute of Standards and Technology, USA, for quality assurance and quality control. This material was analyzed in parallel, in duplicate, with an average analyte recovery inside the range accepted by the NS&T [83]. The same occurred in the analyte recovery in spiked blanks and matrices (67–115%). Analytes in laboratory blanks were subtracted from the samples. Analyte quantification was performed using a nine-level analytical curve following the internal standard procedure. All solvents were residue-analyzed grade from JT Baker. Standard solutions were from AccuStandard. Surrogate recoveries were acceptable and presented mean ± standard deviation = 97 ± 7. Method limits of quantification (LQ) ranged from 1.02 ng.g^-1^ to 8.5 ng.g^-1^ dry weight (dw).

### Analytical methods for microbiological analyses

Cloacal, oral, ocular and tracheal swabs were carefully collected in order to avoid external contamination and accommodated in Cary-Blair media for transportation.

Samples were enriched with Alkaline Peptone Water (APW) containing 1% sodium chloride (NaCl) (37°C/18-24 hours). Samples were then streaked onto Thiossulfate Citrate Bile Salts Sucrose Agar (TCBS) and onto Glutamate Starch Phenol Red Agar (GSP) and incubated to 37°C over night (model 31483, Thelco). Suspected colonies were transferred to Kligler Iron Agar, Lysine Iron Agar and Nutrient Agar with 1% NaCl. Posteriorly, biochemical tests were performed in order to identify species from the Vibrionaceae and Aeromonadaceae families, according to Noguerola & Blanch (2008) [84] and Janda & Abbott (2010) [85], respectively.

### Statistical analyses

Statistical analyses were performed using the SPSS Statistics 17.0 software (IBM). Results from metal, organochlorine and bacteriological analysis were analyzed separately. Basic descriptive statistics was conducted. Data were tested for normal distribution using the Shapiro-Wilk’s test. Since most data were not normally distributed, non-parametric tests were used. Spearman’s rank correlation coefficient was used to verify the strength of the association between the concentration of the same metal in hepatic and muscular tissue and to verify the strength of the association in interelement relationship. A p-value of less than 0.05 was used to indicate statistical significance. For bacteriological analyses, the frequencies of isolation of *Vibrio* spp. and *Aeromonas* spp. were calculated, as well as the frequency of isolation of each species.

## Competing interests

The authors declare that they have no competing interests.

## Authors’ contributions

MDC conceived of the study, carried out metal and statistical analyses and drafted the manuscript; JFM and DCT collected samples in the field, carried out necropsies and helped in several steps, including metal and statistical analyses and in the drafting of the manuscript; RAG helped in the metal analyses; FIC and RCM carried out the organochlorine compound analyses; EMR, RLS and DPR carried out the microbiological analyses; SS conceived the study, participated in its design and coordination and helped draft the manuscript. All authors read and approved the final manuscript.
